# Current perspectives in the management of sepsis and septic shock

**DOI:** 10.3389/fmed.2024.1431791

**Published:** 2024-08-15

**Authors:** Luis Chiscano-Camón, Adolf Ruiz-Sanmartin, Ivan Bajaña, Juliana Bastidas, Rocio Lopez-Martinez, Clara Franco-Jarava, Juan José Gonzalez, Nieves Larrosa, Jordi Riera, Xavier Nuvials-Casals, Juan Carlos Ruiz-Rodríguez, Ricard Ferrer

**Affiliations:** ^1^Intensive Care Department, Vall d'Hebron University Hospital, Vall d'Hebron Barcelona Hospital Campus, Barcelona, Spain; ^2^Shock, Organ Dysfunction and Resuscitation Research Group, Vall d'Hebron Research Institute (VHIR), Vall d'Hebron University Hospital, Vall d'Hebron Barcelona Hospital Campus, Barcelona, Spain; ^3^Departament de Medicina, Universitat Autonoma de Barcelona, Barcelona, Spain; ^4^Immunology Department, Vall d'Hebron University Hospital, Vall d'Hebron Barcelona Hospital Campus, Barcelona, Spain; ^5^Microbiology Department, Vall d'Hebron University Hospital, Vall d'Hebron Barcelona Hospital Campus, Barcelona, Spain

**Keywords:** septic shock, precision medicine, personalized treatment, treatable trait, hemoadsorption, cytokine storm, sepsis phenotypes

## Abstract

Within patients with sepsis, there exists significant heterogeneity, and while all patients should receive conventional therapy, there are subgroups of patients who may benefit from specific therapies, often referred to as rescue therapies. Therefore, the identification of these specific patient subgroups is crucial and lays the groundwork for the application of precision medicine based on the development of targeted interventions. Over the years, efforts have been made to categorize sepsis into different subtypes based on clinical characteristics, biomarkers, or underlying mechanisms. For example, sepsis can be stratified into different phenotypes based on the predominant dysregulated host response. These phenotypes can range from hyperinflammatory states to immunosuppressive states and even mixed phenotypes. Each phenotype may require different therapeutic approaches to improve patient outcomes. Rescue strategies for septic shock may encompass various interventions, such as immunomodulatory therapies, extracorporeal support (e.g., ECMO), or therapies targeted at specific molecular or cellular pathways involved in the pathophysiology of sepsis. In recent years, there has been growing interest in precision medicine approaches to sepsis and phenotype identification. Precision medicine aims to tailor treatments to each individual patient based on their unique characteristics and disease mechanisms.

## 1 Introduction

**Figure 1:**
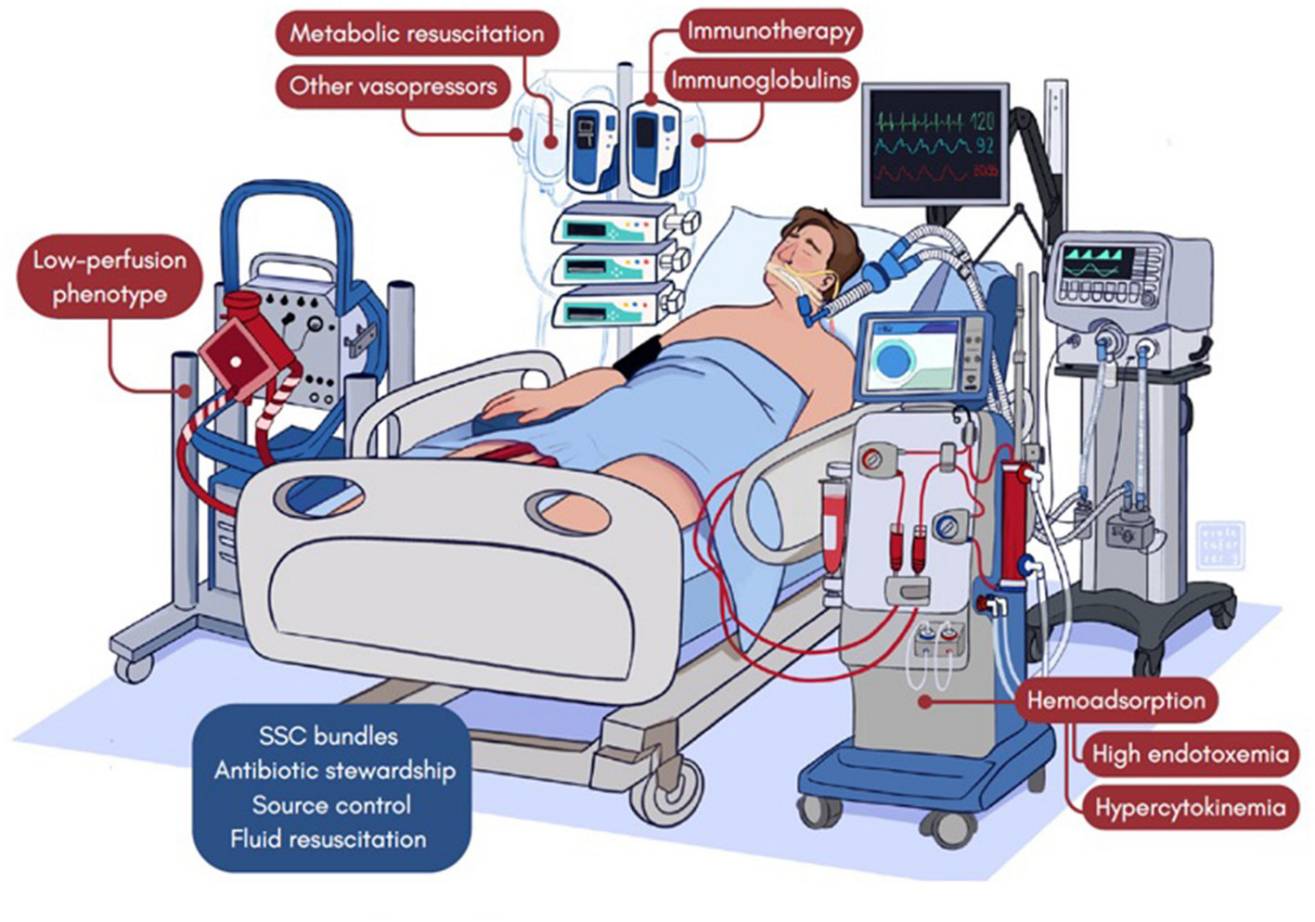
Treatable traits in septic patients.

Sepsis, as defined by the current consensus (1), encompasses a broad range of conditions and can vary in its presentation and severity (2). Not all patients respond to conventional therapy, requiring additional adjunctive therapies. Due to sepsis heterogeneity, response to adjunctive therapies is also heterogeneous with a variety of effects depending on specific endotypes and phenotypes, therefore some individuals may require additional or alternative treatments. Identifying specific subgroups of patients who do not respond to conventional therapy is crucial for developing targeted interventions. Over the years, researchers have made efforts to categorize sepsis into different subtypes based on clinical characteristics, biomarkers, or underlying mechanisms (3). By doing so, they aim to better understand the disease and identify potential treatment options for patients who fall into these subgroups. For example, sepsis can be stratified into different phenotypes based on the predominant dysregulated host response. These phenotypes may include even from hyperinflammatory to immunosuppressed, or mixed phenotypes. Each phenotypes may require distinct therapeutic approaches to improve patient outcomes. Personalized strategies for septic shock can encompass various interventions, such as immunomodulatory therapies, extracorporeal support [e.g., hemadsorption (HA) or extracorporeal membrane oxygenation (ECMO)], or targeted therapies based on specific molecular or cellular pathways involved in sepsis pathogenesis. In recent years, there has been growing interest in precision medicine approaches for sepsis and identifying phenotypes. Precision medicine aims to tailor treatments to individual patients based on their unique characteristics and disease mechanisms (4) (Table 1, Figure 1). In the current body of literature, a range of terms have been utilized to describe presentations of septic shock, particularly those indicating a poor prognosis. In critically ill adult patients, commonly used terms include “refractory septic shock,” “catecholamine resistance,” or “high dose norepinephrine.” However, it is crucial to highlight that there is presently no universally agreed-upon consensus regarding exact definitions for these medical situations (5). Distinguishing between phenotypes in patients with severe septic shock based on identifiable treatable traits, holds the potential to improve patient outcomes.

**Table 1:** Treatable traits in septic patients.

**Treatable Trait**	**Description**	**Biomarker**	**Treatment**
**Hyperinflammatory phenotype**	High endotoxemic phenotype	EAA 0.6 – 0.9	Endotoxin HA devices (e.g.: Toraymixin ®)
	High cytokine phenotype	IL-6 plasmatic concentration, (however *there is still no threshold plasma cytokine level used for beginning or closure of therapy)*	Cytokine HA devices (e.g.: Cytosorb ®)
	High EAA and high cytokine phenotype	Same tresholds as above	Sequential HA *Endotoxin HA with PMX, Toraymixin ®, and subsequent cytokine HA with Cytosorb® has been applied in highly selected patients. Sequential HA is intended to remove the primary stimulus that induces the dysregulated inflammatory response*.
	Macrophage activation-like syndrome (MALS)	Ferritin > 4,420 ng/mL	Anti-IL1 (Anakinra) Anti-TNFα
**Hypoinflammatory phenotype**	Hypogammaglobulinemia	IgG < 500 mg/dL or 2 standard deviations below reference values for age IgM < 35mg/dl	Polivalent IVIG IgM and IgA-enriched polyclonal IVIG dose of 250 mg/kg/d by a 10-h infusion, for 3 consecutive days
	Immunoparalysis T-cell exhaustion syndrome	HLA-DR expression in circulating monocytes <5,000 antibodies bound/cell Ferritin < 4,420 ng/mL	rhIFN_γ_ Monoclonal antibodies (Nivolumab)
**Catecholamine resistant hypotension (CRH)**	Defined as a decreased vascular responsiveness to catecholamine independently of the administered NE dose (5)	Isolated NE > 0.5 μg/kg/min for a minimum of 6 h, to maintain a MAP between 55–70 mmHg.	1) Corticosteroids *[Hydrocortisone (200 mg/day)]* 2) Vasopressin (VP) infusion and titration 3) Metabolic resuscitation *[Ascorbic acid 1,500 mg/6h (15 doses) plus Thiamine 200 mg/12h]*
		CED^*^> 0.25 μg/kg/min and high Renin plasmatic concentrations (n.v. = 2.13–58.78 pg/ml)	Angiotensin-II (AT-II) infusion and titration
**Low-Flow phenotype**	Patients with septic cardiomyopathy exhibiting signs of inadequate perfusion despite the administration and titration of vasoactive drugs (Dobutamine) and receiving supportive treatment guided to other phenotypes.	Lactate plasmatic concentrations and ΔLactate SvcO_2_ < 70 mmHg ΔAvCO_2_ > 6mmHg Echocardiographic parameters of cardiac dysfunction, mainly biventricular failure	Veno-arterial ECMO
**Endothelial dysfunction**	Endothelial cells amplify the immune response and activate the coagulation system. They are both a target and source of inflammation and serve as a link between local and systemic immune responses.	BioADM (>108 pg/mL) MR-proADM (decline in blood plasma concentration to 1.65 nmol/L within 48 h of admission) sTREM-1 (>532 pg/mL)	Adrecizumab Future research (high-dose Nangibotide)

AT-II, angiotensin II; BioADM, circulating bioactive adrenomedullin; CED, catecholamine equivalent dose; CRH, catecholamine resistant hypotension; E, epinephrine; EAA, endotoxin activity assay; ECMO, extracorporeal membrane oxygenation; HA, hemoadsorption IL. IL; Ig, immunogloblulin; IVIG, intravenous immunoglobulin; MALS, macrophage activation-like syndrome; MAP, median arterial pressure; MR-proADM, mid-region proadrenmedullin; NE, norepinephrine; n.v., normal values; PMX, polimyxin; rhIFN_γ_, recombinant human inferteron-gamma; Soluble Triggering Receptor Expressed on Myeloid Cells 1 (sTREM-1); VP, vasopressin. ^*^CED (catecholamine equivalent dose); E = NE = 0.1 μg/kg/min; VP 0.04 U/min = NE 0.1 μg/kg/min.

## 2 Hyperinflammatory phenotype

### 2.1 Hyperinflammatory with high endotoxemic phenotype

Endotoxin has been considered as one of the therapeutic objectives for sepsis and septic shock. Removing endotoxin through blood purification techniques and, specifically, by HA has been suggested (6). Endotoxemia and the overproduction of inflammatory mediators, in the form of a cytokine storm, are associated with the severity of sepsis and septic shock and determine prognosis (7). The Euphrates trial (8) is the largest randomized controlled trials (RCT) conducted. This trial focused on analyzing 450 critically ill patients who were experiencing septic shock and had an endotoxin activity assay (EAA) level ≥0.6. The intervention in this trial consisted of two treatments of PMX hemoperfusion, each lasting 90–120 min, in addition to standard therapy. These treatments were administered within 24 h of enrollment for a group of 224 patients. The remaining 226 patients received sham hemoperfusion along with standard therapy. The results of the Euphrates trial indicated that PMX hemoperfusion did not show a significant difference in 28-day mortality when compared to the control group. However, Klein et al. (9) conducted a *post hoc* study involving 194 patients from the trial who had EAA values ranging from 0.6 to 0.89. In this subgroup analysis, it was observed that patients who received therapy with PMX showed an improvement in survival compared to those who did not receive the treatment. Recently, Shoji et al. (10), in a review of recent studies, demonstrated a survival benefit of PMX hemoperfusion. Lately, in a multicenter, prospective and observational study, it was concluded that the baseline EAA may predict the outcome of critically ill septic patients receiving PMX-HA (11). Osawa et al. (12) on the basis that characteristics of patients with sepsis likely to benefit from PMX-HA are not well known, identified 1,911 patients with sepsis from the JSEPTIC-DIC study and 286 patients with endotoxemic septic shock from the EUPHRATES trial, and concluded that abnormal coagulation (INR > 1.4) and hyperlactatemia in septic patients with high endotoxin activity appear to be helpful to identify patients who may benefit most from PMX-HA. The findings of the EUPHRATES trial introduced two additional factors to consider when selecting patients for endotoxin removal. Firstly, subsequent research revealed that the severity of organ failure plays a crucial role in determining patient response (13). Secondly, certain patients may not experience benefits due to an excessively high burden of endotoxin (14). In this direction, currently, the TIGRIS study (NCT03901807), a prospective, multicenter, randomized open-label trial, is investigating the effects of standard medical care plus polymyxin-based HA vs. the standard care of treatment on subjects with septic shock, multiorgan dysfunction (MODS > 9) and endotoxemia within the range of ≥0.60 to 0.89. It seems reasonable to consider patients with septic shock and severe multiorgan dysfunction, abnormal coagulation and hyperlactatemia who have had adequate control of the infectious focus and have an EAA of 0.6–0.89 as potential candidates for endotoxin HA.

### 2.2 Hyperinflammation with high cytokine phenotype

The use of HA therapy as a personalized therapy may be considered in a situation of septic shock and multiorgan dysfunction refractory to standard treatment. CytoSorb® therapy may be of benefit in conditions characterized by excessive cytokine release because the device effectively attenuates circulating cytokine concentrations during systemic inflammation in humans *in vivo* (15). Other devices have been also studied. HA330 hemoperfusion have shown promising results (16). Also thepeutic plasma exchange (TPE) can remove inflammatory mediators and benefit patients but there is still a path to traverse (17). So, cytokine HA may have a role as a therapy in a particular subgroup of patients with severe septic shock, hyperlactatemia, multiorgan failure, and very high hypercytokinemia (4). Recently, *best practice* criteria have been released for initiating hemoadsorptive therapy in cases of septic/vasoplegic shock that do not respond to standard care and exhibit significantly elevated soluble markers of inflammation. Also, the therapy should commence within 12 h of the diagnosis of septic/vasoplegic shock but no later than 24 h (18). But still to date, there is no threshold plasma cytokine level used for beginning or closure of therapy. Among the studies published to date where HA has been used within the distributive shock patient, it is important to highlight those that are performed on the patient of septic origin and those that account for the plasma concentration of IL-6. Nine of them have reported the plasma concentration of IL-6. Some have used it as an inclusion criterion and others have reported it merely for informational purposes (Table 2).

**Table 2:** Studies that have reported plasmatic concentration of IL-6.

	**IL-6 plasmatic concentration**	**Study summary or main findings**
**Kobe et al**. **(****19****)**	Treatment group: 23,300 (26,500) pg/ml.	7 patients Direct hemoperfusion (CYT-860, CYT-860-DHP) Different clinical criticall ill conditions with SOFA score of 12.93 (4.3).
**Schadler et al**. **(****20****)**	Treatment group: [162–874] pg/ml Control group: 590 [125–2,147] pg/ml.	Randomized, controlled, open-label and multicentric. 97 IMV patients who had severe sepsis or septic shock and ALI. Two groups: one receiving therapy with CytoSorb® hemoperfusion for 6 h per day for up to 7 consecutive days, and the other group receiving no hemoperfusion. Significant elimination of IL-6, ranging from 5% to 18% per blood pass throughout the entire treatment period. However, they did not observe any statistically significant differences in the secondary outcomes, such as the multiple organ dysfunction score, ventilation time, and time course of oxygenation.
**Friesecke et al**. **(****21****)**	Treatment group: 25,523 (1,052–491,260) pg/ml.	20 consecutive patients Refractory septic shock and CytoSorb® treatment was started after 7.8 (3.7) h of shock therapy. Noradrenaline dose could be significantly reduced after 6 (-0.4 μg/kg/min; *p =* 0.03) and 12 h (-0.6 μg/kg/min; *p =* 0.001).
**Schittek et al**. **(****22****)**	Treatment group: 5,000 (939–5,000) pg/ml. Control group: not measured	HA in septic shock with sepsis-associated AKI clinical picture. 76 patients They observed in patients treated with HA a shorter LOS and shorter therapeutic support such as catecholamine dependency and duration of RRT. However, in multivariate analysis (logistic regression for mortality, competing risk for length of stay), they found no significant differences.
**Mehta et al**. **(****23****)**	Treatment group: 1,962.04 (229.09) pg/ml.	Septic shock, 100 patients whom 40 patients survived. In the survivor group, a remarkable reduction of biomarkers levels; PCT (65%, *P* = 0.5859), CRP (27%, *P* = 0.659), serum lactate (27%, *P* = 0.0159) and bilirubin (43.11%; *P* = 0.0565) were observed from baseline after CytoSorb® therapy. The vasopressors dosage remarkably decreased though it was not statistically different; 34.15% (*P* = 0.0816) forE, 20.5 % for NE (*P* = 0.3099) and 51% (*P* = 0.0678) forVP. A significant reduction in inflammatory markers; ILIL 6 and ILIL 10; (87% and 92%, *P* < 0.0001) and in tumor necrosis factor (24%, *P* = 0.0003) was also seen.
**Garcia et al**. **(****24****)**	Treatment group:: 23,897 (23,179) pg/ml Control group: 26,543 (21,373) pg/ml.	Prospectively patients fulfilled refractory septic shock, IL-6 ≥ 1,000 ng/l and a vasopressor dependency index ≥ 3, despite adequate volume resuscitation. 96 matched patients (48 treated with cytokine adsorption, 48 treated without). Cytokine adsorption was provided for three consecutive 24-h sessions initiated within 24 h from shock onset. Within the 72-h intervention period, circulating IL-6 levels (*p =* 0.254) and vasopressor requirements (*p =* 0.555) decreased irrespective of cytokine adsorption use. Intensive care mortality was more pronounced in patients treated with cytokine adsorption than in the control group (control: 20 (42%), cytokine adsorption: 32 (67%), *p =* 0.024) as evidenced by a competing risks hazard ratio for mortality of 1.82 (95% confidence interval, 1.03–3.2; *p =* 0.038).
**Scharf et al**. **(****25****)**	Cytosorb® treatment: 60,529 (10,108–84,000,000) pg/ml. No-Cytosorb®: 25,660 (10,051–600,000) pg/ml.	Retrospectively patients with an IL-6 > 10,000 pg/ml. No difference in IL-6 reduction, hemodynamic stabilization, or mortality in patients with Cytosorb ® treatment compared to a matched patient population. However the underlying diseases resulting in hypercytokinemia were much varied, being separated as sepsis (different reasons except urosepsis) (21.0%), urosepsis (15.2%), septic shock (15.2%), ARDS (13.3%), hemorrhagic shock (8.6%), pneumonia (6.7%), polytrauma (4.8%), and others (15.2%).
**Paul et al**. **(****26****)**	Treatment group: 889.15 (1,307.43) pg/ml	Prospective, real time, investigator initiated, observational multicenter study, patients admitted to the ICU with sepsis and septic shock. 45 patients were included and SOFA score was 12.90 (4.02). In the survivor group, the percentage dose reduction in vasopressor was norepinephrine (51.4%), epinephrine (69.4%) and vasopressin (13.9%) and a reduction in IL-6 levels (52.3%) was observed in the survivor group.
**Hawchar et al**. **(****27****)**	4,240 (0->10^7^) pg/ml.	Cytosorb® registry, 1,434 patients. Indications for HA were sepsis/septic shock (*n* = 936); cardiac surgery perioperatively (*n =* 172); cardiac surgery postoperatively (*n =* 67) and “other” reasons (*n =* 259). At the end of HA, 80.6% of patients were alive. However, there was no significant difference in the predicted and actual hospital mortality. Just as in the whole cohort both the cardiovascular and the pulmonary subscores improved significantly and changes could be determined for CRP in 67.5%, PCT in 45.5% and IL-6 in 20.0% of patients.

Description of the most relevant findings. ALI, acute lung injury; AKI, acute kidney injury; ARDS, acute respiratory distress syndrome; CRP, C-reactive protein; E, epinephrine; HA, hemoadsorption; ICU, intensive care unit; IL, IL; IMV, invasive mechanical ventilation; NE, norepinephrine; PCT, procalcitonin; SOFA, sequential organ failure assessment; VP, vasopressin.

Extremely high hypercytokinemia is an early and unrecognized feature in patients with sepsis, which represents the severe end of the hypercytokinemia spectrum. From a clinical perspective, it is characterized by refractory septic shock, multi-organ dysfunction and very high mortality.

In this scenario, blood purification techniques can blunt the inflammatory process with a rapidly considerable, nonselective effect on the cytokine storm, potentially translating into survival benefit for the patient (28).

### 2.3 Sequential hemoadsorption

In sepsis and septic shock, endotoxemia (presence of endotoxins in the bloodstream) and excessive production of inflammatory mediators, known as a cytokine storm, play a crucial role in the severity of the condition and its prognosis. The goal of sequential HA is to target both endotoxins and cytokines to restore immune homeostasis and alleviate the dysregulated inflammatory response. Current practice has showed that HA helps recovery of immune homeostasis. However, in particular patients, endotoxin-only adsorption may be insufficient (29). Endotoxemia and the overproduction of inflammatory mediators, in the form of a cytokine storm, are associated with the severity of sepsis and septic shock and determine prognosis (7). Sequential HA (endotoxin HA with PMX, Toraymixin ®, and subsequent cytokine HA with Cytosorb®) has been applied in highly selected patients (30). Precision medicine has allowed for a better selection of individuals according to their phenotypic profile to identify patients who could benefit from sequential hemadsorption (cytokine and endotoxin HA). Sequential HA is intended to remove the primary stimulus that induces the dysregulated inflammatory response. The candidates for sequential HA could be patients with refractory septic shock, multiorgan dysfunction, high endotoxemia, and hypercytokinemia (extremely high levels of IL-6). Realtime monitoring of plasma cytokines (IL-6 and IL-10) can guide clinicians to withhold therapy (31).

## 3 Hypoinflammatory phenotype

### 3.1 Hypogammaglobulinemia

The altered mechanism in the adaptive immune system refers to the role of antibodies and immunoglobulins (Ig) (32). Hypogammaglobulinemia, a condition characterized by low levels of Ig, has been associated with higher mortality in sepsis. As a result, it has been proposed as a potential marker for identifying a subgroup of patients who may benefit from immunoglobulin treatment (33). Although the definition of hypogammaglobulinemia is not established, low concentrations of gammaglobulins (IgG) can be defined as values <500 mg/dL in individuals older than 5 years or 2 standard deviations below reference values for age and immunoglobulin concentrations below 300 mg/dL for IgG1, 35 mg/dL for IgM and 150 mg/dL for IgA were associated with shorter survival times (34).

Polyvalent intravenous Ig are a logical approach to modulating both pro- and anti-inflammatory responses in sepsis (35). In the case of adult patients with sepsis, the use of IgM/A-enriched intravenous immunoglobulin has shown positive outcomes. A recent meta-analysis, which included 19 trials and over 1,500 patients, demonstrated a significant reduction in mortality when IgM- and IgA-enriched intravenous immunoglobulin was compared to human albumin solution or no treatment (36).

However, the criteria for determining eligibility for polyvalent intravenous immunoglobulin treatment and the optimal treatment strategy still require further clarification. Currently, one of the recommendation is to administer a single dose of polyclonal IgG at a dosage of 1 or 2 g/kg (level of evidence 2C) (37). Other strategies propose IgM and IgA-enriched polyclonal IVIg dose of 250 mg/kg/d by a 10-h infusion, for 3 consecutive days (38), or an infusion of 42 mg/kg body weight of IgM-enriched polyclonal IVIg once daily for 5 consecutive days (39).

It should be noted that standard administration of intravenous immunoglobulin in patients with sepsis is not recommended according to the 2021 Surviving Sepsis Campaign (SSC) guidelines, thus the recommendation is done for normoglobulinemic patients. Patients with hypogammaglobulinemia, on the other hand, may have a better response to treatment and could potentially benefit from immunoglobulin therapy. Further studies, particularly in patients with hypogammaglobulinemia, are necessary to gather more evidence in this area (39).

## 4 Precision immunotherapy in sepsis. Counterbalancing hyperinflammatory and hypoinflammatory phenotypes

Sepsis is a complex syndrome with diverse manifestations, and patients can present with varying degrees of immune dysregulation. The extremes of immune dysregulation are characterized by hyperinflammation, often referred to as a cytokine storm, and immune paralysis. Both of these states can have detrimental effects on short- and long-term outcomes in sepsis. Hyperinflammation is associated with an excessive and uncontrolled immune response, leading to widespread tissue damage and organ dysfunction. On the other hand, immune paralysis involves a state of immunosuppression where the immune system fails to mount an effective response against the infection, leaving the patient susceptible to secondary infections and complications. The challenge in developing effective immunomodulatory therapies lies in identifying the appropriate timing and targeting of interventions based on the specific immune dysregulation phenotype exhibited by individual patients. A one-size-fits-all approach may not be effective due to the heterogeneity of sepsis.

Patients in a hyperinflammatory state face a heightened risk of mortality within the first 10 days. They exhibit an excessive production of IL (IL)-1b by tissue macrophages, resulting in pancytopenia, bone marrow hemophagocytosis, liver dysfunction, and disseminated intravascular coagulation (DIC). This combination of symptoms is referred to as macrophage activation syndrome (MAS) (40). By utilizing the HS score (41) and the criteria proposed by Shakoory et al. (42), it has been suggested that manifestations of MAS may be present in approximately 3.7% to 4.3% of all sepsis patients, which have been termed as macrophage activation-like syndrome (MALS). Concentrations of ferritin >4,420 ng/mL have a specificity of 98.0% and a negative predictive value of 97.2% in diagnosing MALS (43). *Post hoc* analysis of a RCT demonstrated that the subset of patients with MALS experienced improved survival outcomes when treated with the recombinant antagonist of the IL-1 receptor, anakinra (43).

The opposite end comprises individuals who exhibit immunoparalysis (44). These patients experience an exhausted state of the immune system, which renders them susceptible to secondary infections, prolonged hospitalization, and increased mortality. The reduction in the expression of human leukocyte antigen (HLA)-DR on the surface of circulating monocytes has been proposed as the characteristic feature of immunoparalysis (45). Exploratory investigations have indicated that this condition can be reversed by recombinant human interferon-gamma (rhIFNg) (46), growth factors (G-CSF and GM-CSF), Thymosine alpha 1, Recombinant human IL 7 (rh-IL7: CYT107) and Programmed cell death 1 (PD-1)/Programmed Death Ligand 1 (PDL1) (47–49). Since only a minority of patients demonstrate immunoparalysis (probably no more than 25%-30%), it is futile and potentially hazardous to administer rhIFNg to all sepsis patients.

The PROVIDE RCT has been conducted recently and identified three distinct immune response classifications in sepsis. MALS and immunoparalysis are proposed as stratification criteria for personalized adjuvant immunotherapy (50). It was a study conducted using a double-blind, double-dummy randomized clinical design. A total of 240 patients diagnosed with sepsis caused by lung infection, bacteremia, or acute cholangitis were enrolled. The study involved measuring serum ferritin levels and HLA-DR/CD14 expression. Patients exhibiting features of macrophage activation-like syndrome (MALS) or immunoparalysis were randomly assigned to receive treatment with anakinra, recombinant interferon-gamma (rhIFN_γ_), or placebo. The primary outcome measured was mortality at 28 days, while the secondary outcome was the classification of sepsis immune response. By utilizing biomarkers such as ferritin >4,420 ng/mL and <5,000 HLA-DR receptors/monocytes, patients were categorized into MALS (20.0%), immunoparalysis (42.9%), and intermediate (37.1%) groups. Mortality rates were found to be 79.1%, 66.9%, and 41.6%, respectively. Survival at 7 days with a decrease in the Sequential Organ Failure Assessment (SOFA) score was achieved in 42.9% of patients in the immunotherapy arm compared to 10.0% in the placebo arm (*p* = 0.042).

IL-1β is a key cytokine at the interface of innate and adaptive immunity. It is produced by several types of myeloid cells upon stimulation by PAMPs and DAMPs (51). The binding of mature IL-1β to its cognate receptor IL-1R1 on nearby cells and subsequent formation of a ternary signaling complex with IL-1RAcP triggers a proinflammatory response with the production of inflammatory mediators, such as IL-6 (52). The inflammatory response involving interleukin-1β (IL-1β) has been thought to play an important role in the development of late-phase sepsis. Recently, Hommet et al. (53) described the discovery of a low-molecular weight human-IL-1β antagonist that blocks the interaction with the IL-1R1 receptor demonstrating the relevance for future development of hIL-1β directed therapeutics.

Regarding anti-TNF-α therapy for patients with sepsis a recent meta-analysis (54) included seventeen studies with a total of 8,971 patients and when all forms of anti-TNF-α therapy were pooled together, there was a significant reduction of 28-day all-cause mortality with respect to placebo and individually the subgroup analysis showed that anti-TNF-α antibodies (monoclonal and polyclonal) reduced mortality (OR = 0.90, 95% CI: 0.81–0.99; *p* = 0.04). Additionally, there was a trend toward better survival in patients with high levels of IL-6 (>1,000 pg/ml) and patients with shock if they were treated with anti-TNF-α therapy (OR = 0.85, 95% CI: 0.72–1.00; OR = 0.80, 95% CI: 0.62–1.04).

Among other markers which are still under investigation, mentioning both programmed cell death protein 1 (PD-1 and PD-L1) whom are type I transmembrane immunoglobulin (Ig) superfamily members. Following antigen clearance and the resolution of the inflammation, the PD-1 receptors on the surface of cytotoxic T lymphocytes (CTLs) bind to their ligands, PD-L1 and PD-L2, to generate a co-inhibitory signal, which suppresses the CTL expansion (55). The recently developed immune checkpoint blockers (ICBs) can counteract this cancer-induced ligand-receptor association, enabling reinvigoration of exhausted CTLs, restoration of anticancer immunity and suppression of cancer growth (56). Recent clinical studies of the PD-1 inhibitor antibody, Nivolumab (Opdivo®), first approved for the treatment of melanoma, demonstrated favorable safety and tolerability in the treatment of septic patients (57).

## 5 Catecholamine–resistant-hypotension

### 5.1 Focus on vasopressin and angiotensin-II

Systemic vasodilatation and arterial hypotension are landmarks of septic shock. Whenever fluid resuscitation fails to restore arterial blood pressure and tissue perfusion, vasopressors agents are necessary. Norepinephrine, a strong α-adrenergic agonist, is the standard vasopressor to treat septic shock-induced hypotension but adrenergic vasopressors have been associated with several detrimental effects, including organ dysfunction and increased mortality (58, 59).

The renin-angiotensin-aldosterone system (RAAS) provides an important physiologic mechanism to prevent systemic hypotension under hypovolemic conditions, such as unresuscitated septic shock (60). In addition to its classical hemodynamic function of regulating arterial blood pressure, angiotensin-II (AT-II) plays a key role in several biological processes, including cell growth, apoptosis, inflammatory response, and coagulation. It may also affect mitochondrial function (61, 62).

The administration strategy of vasopressors in distributive shock, particularly the impact of early and multimodal administration, is an area of interest due to its potential significant impact on outcome. When to initiate a second (or third) line vasopressor has long been debated. Most of the data available arises from the use of vasopressin (63). Vasopressin is recommended by the SSC for adults with septic shock who have inadequate mean arterial pressure (MAP) despite low to moderate doses of norepinephrine, but with a weak recommendation due to moderate quality evidence. The suggestion to use vasopressin primarily stems from subgroup analyses of randomized trials and observational studies, which suggest better outcomes when vasopressin is initiated in less severe patients or those receiving lower doses of norepinephrine. In the VASST trial comparing the combination of norepinephrine and vasopressin to norepinephrine alone, patients who received <15 μg/min of NE showed better survival rates with the addition of vasopressin (64). The final idea or one proposed mechanism for the improved survival with lower-dose norepinephrine and vasopressin combination is a reduction in catecholamine exposure (65).

AT-II is a natural hormone with endocrine properties, autocrine and paracrine effects recently approved by the USA Food and Drug Administration (FDA) for the treatment of distributive shock thus it has vasoconstrictor effects at both arterial and venous levels (66). Firstly, data from a study by Chawla et al. (67) supports the safety and efficacy of AT-II in the treatment of patients with “Cathecolamine Resistant Hypotension” (CRH). The rates of adverse events of special interest were similar in the ATII and placebo groups in that pilot study. Specifically, rates of tachyarrhythmias, distal ischemia, ventricular tachycardia, and atrial fibrillation were similar in the two groups. In the ATHOS-3 trial (68) 344 patients refractory to catecholamine treatment with norepinephrine (0.2 μg/kg/min or equivalent) were randomized to receive AT-II or placebo. The main objective of the study was to achieve an increase in baseline MAP ≥ 10 mmHg or raise arterial pressure >75 mmHg; this was achieved in 69.9% of ATII patients and in 23.4% of the placebo group without significant differences in side effects. Even, Wieruszewski et al. (69) performed an exploratory *post-hoc* analysis of ATHOS-3 trial and concluded that initiation of AT-II at a low norepinephrine equivalent dose (NED) of ≤ 0.25 μg/kg/min was associated with higher likelihood of survival when compared to placebo. *Post-hoc* subgroup analyses of the ATHOS-3 trial have provided additional data to show that patients on renal replacement therapy (RRT) at randomization had better rates of recovery to RRT independence at day seven and improved survival with AT-II (70) and that patients with an angiotensin I/II (ANGI/II) ratio below the median were significantly more likely to survive (suggesting a degree of AT-II deficiency) (71). Recently, Bellomo et al. (72) observed that renin levels are markedly elevated in vasodilatory shock and could identify patients in whom treatment with ATII may be more beneficial.

Thus, among patients with vasodilatory shock, there was significant heterogeneity of RAAS endotype. Currently, ATII should not be considered as a first-line agent, but having demonstrated its safety and physiological efficacy, it may have a role as a vasopressor adjuvant. In conclusion, the impression according to current evidence, the patient subpopulations that could benefit the most from the use of ATII are those who, despite receiving standard care, are in distributive shock experiencing renal failure requiring replacement therapy and have elevated plasma renin levels and initiate ATII when the dose of vasoactive drugs is still not high (73).

### 5.2 Corticosteroids

Septic shock involves unchecked widespread inflammation, leading to multiple organ failure and potentially death. It is now well-established that the host's failure to properly activate the hypothalamic-pituitary-adrenal axis significantly contributes to the severe systemic inflammation seen in infections. Proinflammatory mediators at inflamed sites counteract the anti-inflammatory response, but this can be countered by administering external corticosteroids. In cases of sepsis, corticosteroids act through both genomic and nongenomic pathways to restore cardiovascular stability, halt systemic and tissue inflammation, recover organ function, and avert death (74). The actual SSC recommendation is toward a suggestion for its use for adults with septic shock and an ongoing requirement for vasopressor therapy mainly with catecholamine equivalent dose of >0.25 μg/kg/min for a minimum of 6 h. There are some RCT and a meta-analysis published lately. Annane et al. (75) evaluated the effect of hydrocortisone-plus-fludrocortisone therapy, drotrecogin alfa-activated and the combination of the three drugs finding 90-day all-cause mortality was lower among those who received hydrocortisone plus fludrocortisone than among those who received placebo and even the number of vasopressor-free days to day 28 was significantly higher in the hydrocortisone-plus-fludrocortisone group than in the placebo group (17 vs. 15 days, *P* < 0.001), as was the number of organ-failure-free days (14 vs. 12 days, *P* = 0.003). Venkatesh et al. (76) randomly assigned patients with septic shock who were undergoing mechanical ventilation to receive hydrocortisone (at a dose of 200 mg per day) or placebo for 7 days or until death or discharge from the intensive care unit and did not result in lower 90-day mortality than placebo, patients who had been assigned to receive hydrocortisone had faster resolution of shock and had a shorter duration of the initial episode of mechanical ventilation. Finally, an updated meta-analysis (77) found systemic corticosteroid to accelerate resolution of shock (MD 1.52 days; 95% CI 1.71–1.32). Through the RECORD trial (78), efforts are being made toward identifying subgroups of patients that would be responsiveness to corticosteroid therapy; there is the hypothesis that community-acquired pneumonia related sepsis, septic shock, or sepsis-related ARDS, bacterial or viral sepsis, may share common signatures.

### 5.3 Metabolic resuscitation and Vitamin C (ascorbic acid)

During septic shock, progression of tissue injury takes place, with mitochondrial dysfunction playing a central role in this process. This impairment of mitochondrial function leads to disrupted energy production and uncoupling of oxidative phosphorylation, resulting in what is commonly referred to as oxidative stress. This oxidative stress is manifested by an elevation in reactive oxygen (ROS) and nitrogen (RNS) species, which inflict harm on the cell membrane, intercellular connections, and endothelial barrier, ultimately damaging the glycocalyx (79). Furthermore, it causes alterations in vascular tone and heightened capillary permeability, along with a certain degree of resistance to the effects of catecholamines (80). Due to their intracellular physiological impacts, the administration of corticosteroids, ascorbic acid, and thiamine has been suggested as a component of adjunctive therapy for sepsis, referred to as “metabolic resuscitation.”

The clinical importance of administering high doses of vitamin C, along with hydrocortisone and thiamine, as a “sepsis cocktail” was made widely known by Marik et al. (81). Their study demonstrated notable reductions in hospital mortality, duration of reliance on vasopressors, and organ damage. An important meta-analysis (82) warrants attention for its examination not only of corticosteroids but also of the “cocktail metabolic resuscitation” (hydrocortisone, vitamin C, and thiamine). The findings did not demonstrate statistical significance in terms of mortality. Nevertheless, they did indicate that the combination of these three substances enhanced organ dysfunction (assessed by ΔSOFA within the initial 72 h of treatment) and decreased the requirement for vasoactive amines. However, a recent study conducted by Lamontagne et al. (83) has demonstrated that the administration of vitamin C in sepsis has detrimental effects, leading to an increase in morbidity and 28-day mortality. This study provided a sophisticated analysis that resolves the lingering uncertainty surrounding the use of vitamin C in septic patients. Nonetheless, there is a particular subgroup of patients with refractory septic shock that warrants attention as they experience high mortality rates and were underrepresented in this study, with <60% of the study population meeting the criteria for septic shock. Interestingly, when vitamin C was administered to this subgroup of critically ill patients, the results were inconclusive. Furthermore, the combined therapy of hydrocortisone, vitamin C, and thiamine, which holds the strongest physiological rationale, was not assessed in this study (84).

These three components of metabolic resuscitation individually or in amalgamation without involving the three substances have not yet demonstrated the anticipated outcomes, but additional considerations should be taken into account when interpreting recent trials and planning future studies: was the dosage of vitamin C sufficient? Should the administration of vitamin C be guided by plasma vitamin C levels? What is the ideal timing for administering vitamin C, and what is the optimal duration? Is there a biomarker that is pertinent to the use of vitamin C? What outcome should be assessed? Which critically ill patients might derive the greatest benefits? (85).

## 6 Low-flow phenotype

We propose the concept of the “low flow phenotype” to encompass patients with septic cardiomyopathy who exhibit signs of inadequate perfusion despite the administration of vasoactive drugs and receiving supportive treatment guided to other phenotypes. The utilization of mechanical circulatory assistance continues to be a subject of controversy when managing refractory septic shock in adult patients (86). The veno-arterial (VA) configuration of ECMO support presents an appealing choice for shock management, particularly in patients experiencing severe concurrent cardiac and pulmonary failure but high-quality evidence supporting its use in adults is still limited. Riera et al. (87) conducted a review highlighting ECMO as a supportive method rather than a treatment, but concluding that in specific cases, with an adequate configuration and a well-defined management, this method may be savior in adult septic patients with no other options for survival. Bréchot et al. (88) ruled a multicentric retrospective study where patients treated with VA-ECMO had more severe myocardial dysfunction, more severe haemodynamic impairment and more severe organ failure than did controls, with *p* < 0.0001 for each comparison, however survival at 90 days for patients treated with VA-ECMO was significantly higher than for controls (60% vs. 25%, risk ratio [RR] for mortality 0.54, 95% CI [0.40–0.70]; *p* < 0·0001). Ling et al. (89) conducted a systemic review including 14 observational studies with 468 patients that concluded that when treated with VA ECMO, most patients with septic shock and severe sepsis-induced myocardial depression survive. However, VA ECMO has poor outcomes in adults with septic shock without severe left ventricular depression. Pooled survival was 36.4% (95% confidence interval [CI]: 23.6%−50.1%). Survival among patients with left ventricular ejection fraction (LVEF) < 20% (62.0%, 95%–CI: 51.6%−72.0%) was significantly higher than those with LVEF > 35% (32.1%, 95%–CI: 8.69%−60.7%, *p* = 0.05).

## 7 Endothelial dysfunction

Sepsis profoundly impacts various aspects of endothelial cell (EC) function and is considered a pivotal factor in the transition from sepsis to organ failure. The diverse endothelial functions affected by sepsis encompass vasoregulation, barrier function, inflammation, and hemostasis. These alterations often involve glycocalyx shedding, leading to anomalies in nitric oxide metabolism, increased generation of reactive oxygen species resulting from the diminished endothelial-associated antioxidant defenses, transcellular communication, proteases, exposure of adhesion molecules, and activation of tissue factor, among other mechanisms (90).

In the inflammatory cascade of sepsis, bacterial components activate both immune cells and the endothelium, triggering the production of cytokines in a self-perpetuating cycle. This activation prompts endothelial cells to express adhesion molecules, facilitating the binding of immune cells. This, in turn, initiates the transmigration of immune cells to the injury site. Reactive oxygen species (ROS) released by both immune cells and endothelium further amplify the inflammatory response. These combined insults result in the shedding of glycocalyx, induction of adhesion molecules, heightened endothelial permeability, and endothelial apoptosis. Chemokines released by immune cells and the endothelium play a role in recruiting immune cells from the bone marrow. The shift in the balance between endothelial nitric oxide synthase (eNOS) and inducible nitric oxide synthase (iNOS) leads to an excessive synthesis of nitric oxide (NO) and subsequent vasodilation (91).

During sepsis, the coagulation cascade is triggered by activated cells of the innate immune system, such as neutrophils and monocytes, resulting in clot formation mainly in the microcirculation, a process known as immunothrombosis. Such a crucial pathophysiological mechanism in sepsis, emerges from the intricate interplay between innate immune responses and endothelial cells, juxtaposed with the involvement of platelets and the coagulation cascade. While initially advantageous for the host, unbridled and systemic activation of this process during sepsis can result in complications such as thrombotic and bleeding issues, ranging from subtle abnormalities in coagulation tests to severe clinical conditions like Disseminated Intravascular Coagulation (DIC). Endothelial dysfunction, marked by glycocalyx degradation, heightened vascular permeability, and the proinflammatory and procoagulant attributes of endothelial cells, further facilitates the progression of immunothrombosis. Future studies are crucial for unraveling the pathophysiological mechanisms underlying immunothrombosis in sepsis, identifying potential prognostic biomarkers, developing risk scores to predict sepsis outcomes, and testing innovative therapeutics against immunothrombosis in sepsis (92).

### 7.1 Adrenomedullin

Within the realm of biomarkers that reflect endothelial dysfunction, we might focus on adrenomedullin (ADM), which is a vasoactive hormone, with reported prognostic and potentially therapeutic value in sepsis. ADM has immunomodulation and endothelial barrier-stabilizing properties, maintaining vascular integrity (93). Its tropism for the vascular endothelium, interstitium and smooth muscle, and its vasodilatory properties, may contribute to sepsis hypotension and increased vascular permeability. At high concentrations, ADM leads to excessive vasodilation, and increased plasma levels of ADM are associated with high vasopressor requirements, multiorgan dysfunction, and mortality (94–96).

Lundberg et al. (97) concluded that circulating bioactive adrenomedullin (bio-ADM) on admission is associated with 30-day mortality and organ failure in sepsis patients as well as in a general ICU population. Elevated bio- ADM was associated with an increased need of vasopressors, OR 1.33 (95% CI 1.23–1.42) (95% CI 1.17–1.50) and even a cut-off of 70 pg/mL differentiated between survivors and non-survivors in sepsis, but a Youden's index derived threshold of 108 pg/mL performed better.

On the other hand, there have been reports of a correlation between a more pronounced reduction in MR-proADM (mid-regional pro-adrenomedullin) levels during the patient's stay in the intensive care unit (ICU) and favorable outcomes. Survivors exhibited a decline in blood plasma concentration to 1.65 nmol/L within 48 h of admission, and their levels remained lower on the 5^th^ day compared to non-survivors. The potential of MR-proADM in promptly identifying severe cases with an elevated risk of organ dysfunction has been assessed, regardless of the source of infection. Additionally, MR-proADM is utilized to assist healthcare professionals in making clinical decisions concerning the allocation of hospital and ICU resources, as it exhibits the highest predictive value for mortality when compared to PCT, CRP, SOFA scores, and lactate (98, 99).

Mentioning it as potential treatable trait, Adrecizumab (HAM8101) is a non-neutralizing anti-ADM antibody with epitope specificity for the N-terminal moiety of ADM. By binding to ADM, adrecizumab does not entirely block ADM function, though it reduces its capacity to elicit a second messenger response, thus, adrecizumab can reduce vasodilation by subtracting excessive levels of interstitially located ADM. The increased net activity of ADM in the blood circulation could promote stabilization of endothelial permeability (100). The AdrenOSS-2 study, a phase 2a double-blind, randomized, placebo-controlled biomarker-guided trial, addressed the safety and tolerability of adrecizumab in patients with septic shock and elevated plasma concentrations of circulating biologically active ADM (>70 pg/ml) (101). Adrecizumab was well tolerated and showed a favorable safety profile. Although it was not the primary objective of the study, an improvement in multi-organ dysfunction was observed. A subsequent study, the ENCOURAGE study, is a phase IIb/III clinical trial that will assess adrecizumab (4 mg/kg) in septic shock patients immediately after initiation of vasopressors. Interestingly, this study combines predictive and prognostic enrichment strategies with the primary objective of reducing 28-day mortality and the SOFA score.

### 7.2 Soluble triggering receptor expressed on myeloid cells 1

Regarding other markers, the Triggering Receptor Expressed on Myeloid Cells (TREM) family includes several isoforms that share low sequence homology with each other and have only one immunoglobulin-like domain. Engagement of TREMs triggers a signaling pathway leading to intracellular calcium mobilization, actin cytoskeleton rearrangement and activation of transcriptional factors. Its expression at the cell-surface of these effector cells is significantly enhanced in skin, biological fluids and tissues infected by Grampositive or Gram-negative bacteria as well as by fungi (102). TREM and is soluble part, serves as diagnosis of septic shock (103) and as a prognostic marker of infection (104) and as a target molecule for adjuvant treatment of sepsis. Nangibotide is an inhibitor of triggering receptor expressed on myeloid cells-1 (TREM-1) (105). A phase 2b clinical trial, ASTONISH, evaluated its efficacy, safety, and tolerability in patients with septic shock, specially focused on the high sTREM-1 subgroup and its inhibition properties (ClinicalTrials.gov Identifier NCT04055909) (106). Still the results were not met in the overall population for the primary outcome, which was the difference in total SOFA score from baseline to day 5, however, in prespecified exploratory analyses limited to those with sTREM-1 concentrations of at least 532 pg/mL, the authors report a significant change in ΔSOFA score at day 5, favoring high-dose nangibotide. Given the prognostic influence of sTREM-1, future studies that rely on a single cutoff of this biomarker for patient selection need to ensure study groups have balanced distributions in absolute concentrations.

### 7.3 Thrombocytopenia-associated multiple organ failure

Thrombocytopenia-associated multiple organ failure (TAMOF) is a clinical phenotype characterized by a range of syndromes linked to widespread microvascular thromboses, such as thrombotic microangiopathies (TMAs), thrombotic thrombocytopenic purpura/hemolytic uremic syndromes (TTP/HUS), and disseminated intravascular coagulation (DIC). TAMOF manifests as a sudden onset of thrombocytopenia that progresses to multiple organ failure in critically ill patients. The reduction in platelet counts indicates their role in the formation of widespread microvascular thromboses, leading to organ ischemia and dysfunction. With the current management strategy, mortalities from TAMOF remain high, ranging from 5% to 80% (107).

Pro-thrombotic and anti-fibrinolytic responses, which are helpful during focal injury, may be injurious in the setting of systemic endothelial injury and are manifested by thrombocytopenia, systemic thrombosis, and multiple organ failure. Critically ill patients develop systemic endothelial microangiopathic disease after many types of systemic insults. The pathophysiology of these thrombotic microangiopathies caused by systemic endothelial injury can be characterized as part of a spectrum of three phenotypes, TTP, consumptive DIC, and non-consumptive secondary TMA (108).

Mounting evidences are suggesting that a nonspecific plasma therapeutic strategy such as TPE may have a role in reversing MOF and improving outcomes in patients with TAMOF. Currently, the American Society for Apheresis gives a category III recommendation—“Optimum role of apheresis therapy is not established. Decision making should be individualized”—for TPE in sepsis with MOF (109). Currently, there is no monotherapy for DIC. Various agents had been tried without success such as heparin, antithrombin III, recombinant tissue factor pathway inhibitor, recombinant activated protein C, protein C concentrate, and recombinant soluble thrombomodulin (110).

## 8 Conclusions

While precision medicine in septic shock is still an evolving field, progress is being made in identifying relevant biomarkers, genetic markers, and clinical tools that can aid in patient classification and treatment decision-making. Ongoing research and clinical trials are exploring the potential of precision medicine approaches, and as our understanding of the disease continues to deepen, precision medicine is likely to play an increasingly important role in optimizing the clinical management of septic shock patients.

Certainly, we acknowledge that treatments based on these phenotypes are not yet supported by evidence in the form of RCTs. Therefore, they are not recommended by the SSC guidelines. We want to emphasize this point conscientiously. However, subgroups of patients refractory to conventional treatments could benefit from specific treatments based on phenotype characterization. These are patients with a high risk of mortality and are often not included in randomized studies. Their accurate identification and selection could lead to an improvement in survival rates.

It's important to note that the implementation of precision medicine in septic shock is an ongoing process, and the availability of specific treatments or personalized therapies tailored to individual patient characteristics may vary. Clinical decisions should always be made in consultation with healthcare professionals who have access to the most up-to-date research and clinical guidelines. Also emphasize that despite differing phenotypes, hemodynamic support requirements may remain consistent, and the same patient may present with multiple phenotypes.

The ultimate goal of precision medicine in septic shock is to develop targeted treatments or personalized therapies that are more effective for specific patient subgroups. By tailoring interventions based on the characteristics of individual patients, clinicians can potentially improve outcomes and reduce the overall burden of the condition.

## Author contributions

LC-C: Conceptualization, Data curation, Formal analysis, Methodology, Project administration, Validation, Writing – original draft, Writing – review & editing. AR-S: Supervision, Validation, Writing – review & editing. IB: Writing – review & editing. JB: Supervision, Writing – review & editing. RL-M: Supervision, Validation, Writing – review & editing. CF-J: Supervision, Validation, Writing – review & editing. JG: Supervision, Validation, Writing – review & editing. NL: Supervision, Validation, Writing – review & editing. JR: Supervision, Validation, Writing – review & editing. XN-C: Supervision, Validation, Writing – review & editing. JR-R: Conceptualization, Data curation, Project administration, Supervision, Validation, Writing – review & editing. RF: Conceptualization, Methodology, Validation, Visualization, Writing – review & editing.
